# Neural substrate of posterior left atrium: A novel modulation for inducibility and remodeling of atrial fibrillation in canine

**DOI:** 10.1371/journal.pone.0176626

**Published:** 2017-05-05

**Authors:** Mu Qin, Li Li, Xu Liu, Tao Liu, Shao Bo Shi

**Affiliations:** 1 Department of Cardiology, Shanghai Chest Hospital Affiliated to Shanghai Jiaotong University, Shanghai, China; 2 Department of Cardiology, the First College of Clinical Medical Sciences, China Three Gorges University, Yichang, China; 3 Cardiovascular Research Institute of Wuhan University, Wuhan, China; University of Minnesota, UNITED STATES

## Abstract

**Background:**

The neural mechanism of posterior left atrium (PLA) for genesis of atrial fibrillation has not been completely elucidated. We sought to assess the contribution of PLA denervation on atrial fibrillation (AF) inducibility and atrial remodeling.

**Methods and results:**

After left thoracotomy in anesthetized dogs (n = 32), electrode catheters were attached to the PLA, left atrial roof, left pulmonary vein and left atrial appendage. Experiment 1 (n = 16): Vagal stimulation (VS group, n = 8) led to more pronounced ERP shortening in PLA than in other sites (CTL:71±7 ms vs VS: 52±6 ms, P<0.05;). Compared with control group (CTL group, n = 8), atropine alone or with propranolol applied to PLA greatly inhibited VS-induced ERP shortening, ERP dispersion increase, and AF inducibility in the left atrium (P<0.05); but ERP was not significantly different between atropine alone and DB conditions (Atro:85±8 ms vs DB:90±9ms, P>0.05). In addition, domain frequency (DF) of VS-induced AF waveform was not affected by atropine alone, while selective double autonomic blockade at PLA significantly decreased DF at all sites (P<0.05). Experiment 2 (n = 16): In group 1 (n = 8), ERP was markedly shortened in the first 2 hours (11–19% decrease) and then stabilized; however, WOV was progressively widened throughout the 6 hours rapid atrial pacing (BS: 51±9ms vs 6th hour: 161±30ms, P<0.05). After drug application, ERP was increased in all sites of atria, the ERP dispersion was significantly decreased (Atro: 2.36±0.02 vs 6th hour: 5.09±0.07, P<0.05) and AF could be induced in only 1 of 8 dogs. In group 2 (n = 8), 6 hours rapid atrial pacing failed to shorten the ERP and increased ERP dispersion, and only 2 episodes of AF could be induced (WOV = 0).

**Conclusion:**

Local denervation of PLA, as predominant atrial autonomic profile, greatly inhibits AF inducibility and atrial remodeling.

## Introduction

The autonomic nervous system facilitates genesis and maintenance of atrial fibrillation (AF)[1–2]. Evidence from clinical data suggested that vagal denervation enhanced benefit of circumferential pulmonary vein (PV) ablation in reducing AF recurrence. Ablation of ganglion plexi (GP), by destroying the autonomic neurons, potentially is more effective in reducing the release of neurotransmitter, and may be more effective to improve PV isolaiton outcomes[3]. However, endocardial GP ablation strategies that target the left atrial (LA) parasympathetic nervous system have been less successful[4], since the interconnected effect of neural network scattered throughout the atria has not been well documented.

Previous studies[5–6] found that the PLA was most richly innervated with nerve bundles, up to one-third located away from fat pad, suggesting that GP ablation strategies targeting atrial fat pad would be unlikely to result in complete PLA denervation. Furthermore, animal study showed that distribution of M_2_R receptors and acetylcholine sensitive K^+^ channels (I_K,Ach_) also was more pronounced in PLA than other LA regions. Vagal stimulation caused greater decrease in ERP in PV and PLA, and the heterogeneity of vagal effects on refractoriness is more pronounced in the PLA as compared with other atrial sites[5].This heterogeneity may increase atrial refractoriness dispersion and AF re-entry susceptibility.

Based on the aforementioned observations, we postulated that selective autonomic nervous system disruption in PLA would modify substrate for AF genesis and maintenance, and examined the effect of pharmacologically induced PLA autonomic blockade on AF inducibility and atrial remodeling.

## Methods

All protocols conformed to the Guide for the Care and Use of Laboratory Animals published by the US National Institutes of Health (NIH Publication No. 85–23, revised 1996). Experiments were approved by the Animal Care and Use Committee of Shanghai Chest Hospital Affiliated to Shanghai Jiaotong University.

### Experiment animals

32 male mongrel dogs weighing 19±5 kg were supplied by the center of experimental animal in agricultural college of Shanghai Jiaotong University (No.2678 Qixin Road, Shanghai, 210101). All the dogs were under one roof and a common airspace, however, each dog had its own kennel space bordered by metal bars, so that animals could interact and see/hear each other while being safely separated. Each kennel had an outdoor area and an indoor area with central heating and a resting place (temperature:10–20°C, humidity: 40–70%, lighting: 12 h per day, 2 animals per pen). The animals were provided various types of chew toys for enrichment. A certified animal handler tended to the dogs every day (feeding, drinking, cleaning etc). Food was withdrawn 24 h before anaesthesia.

### Animal preparation

**Fig 1:**
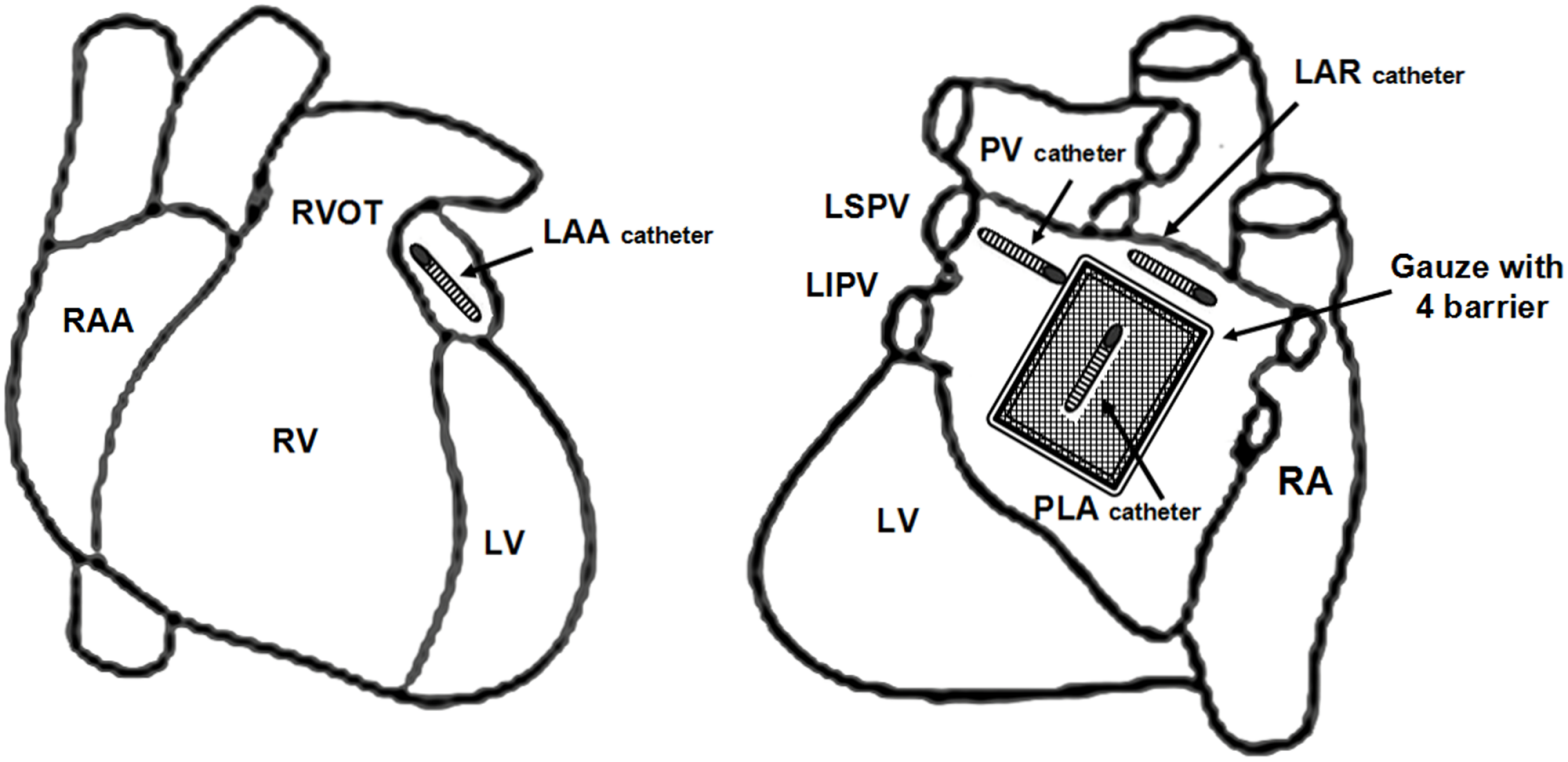
Schematic representation of the catheters and gauze in canine atria. PV, pulmonary vein; PLA, posterior left atrium; LAR, left atrial roof; LAA, left atrial appendage.

Experiments were performed on healthy adult mongrel dogs that were intravenously anesthetized in a posterior lateral small saphenous vein with pentobarbital sodium(30 mg/kg) and mechanically ventilated by a respirator with room air with a positive pressure respirator (Harvard Apparatus Co., Natick, MA, USA). A surgical plane of anesthesia was maintained using 3% pentobarbital sodium throughout the experiment. All efforts were made to minimize suffering. Surface ECG and blood pressure (BP) were continuously recorded. Core body temperature was maintained at 36.5°C±1.5. The chest was entered via left sided thoracotomy at the 4th intercostal space, multielectrode catheters (Biosense Webster, Diamond Bar, CA, USA) were secured for recording at epicardium of the left superior pulmonary vein (PV), posterior left atrium (PLA), left atrial roof (LAR) and left atrial appendage (LAA; Fig 1).

### Stimulation protocol

#### Programmed stimulation

A programmed electrical stimulation (PES) protocol was used to examine atrial effective refractory period (ERP) and AF inducibility. Briefly, PES consisted of an eight-stimulus (S1) drive train followed by a ninth extra stimulus (S2). The CL of the S1 train was 330ms, 10×diastolic threshold. The S1-S2 interval was progressively reduced by 10 ms per cycle until the S2 stimulus could no longer evoke atrial deflection. ERP was defined as the longest S1-S2 interval that caused loss of atrial depolarization. The stimulation was delivered at PV, PLA, LAR and LAA respectively to record ERP of each site.

#### Vagal stimulation

For vagal stimulation, the left cervical vagus nerve was isolated, a bipolar stainless steel electrode was attached to the nerve, and stimulation was performed at 20 Hz (15–20 V, 2–8 ms) (Grass S88, Astromed Inc., Warwick, USA). A vagal response was defined as (1) sinus node slowing by at least 25% or (2) PR prolongation by more than 25% or 2:1 AV block.

#### Rapid atrial stimulation

Rapid atrial pacing was delivered (1200 bpm, 2×threshold, 1 ms duration) at the left atrial appendage for 6 hours. After each pacing hour, rapid atrial pacing was temporarily stopped to measure the ERP and AF inducibility.

#### AF definition

AF was defined as irregular atrial rates faster than 500 beats/minute (bpm) associated with irregular atrioventricular conduction lasting >5s. During ERP measurements, if AF was induced by decremental S1-S2 stimulation, the longest minus the shortest S1-S2 interval at which AF was induced was designated as the width of the window of vulnerability (WOV). The cumulative WOV (ƐWOV) was counted as the sum of the WOV acquired at all sites in each dog.

### Electrogram spectrum analysis

Bipolars electrograms were recorded continuously from electrodes of PV, PLA, LAR and LAA. Power spectrum analysis was used to quantitate characteristics of bipolar electrograms recorded during AF; a 4096-point fast Fourier transformation and Lab Chart7.0 software were used. The electrogram periods were tapered at their edges to a zero value by a Hanning window, rectified, and processed with a 5- to 40-Hz band-pass filter. A dominant frequency (DF) corresponding to the highest peak in the power spectrum was determined for each spectrum.

### Experimental protocols and drug administration

#### Experiment 1

Sixteen dogs were assigned to control (CTL) group (n = 8) and vagal simulation (VS) group (n = 8). ERP and WOV from pulmonary vein (PV), PLA, LA roof (LAR) and LA appendage (LAA) electrodes and AF inducibility and duration were examined in the two groups at baseline. A gauze moistened with atropine (1mM) then was applied under the subepicardium of the PLA region after demarcating four borders, namely LAA base, left inferior PV base, interatrial septum, and atrioventricular groove. To achieve parasympathetic and sympathetic nerve double blockade (DB), atropine (1mM) and propranolol (1mM) were applied after PLA was flushed with saline. ERP,WOV and AF induction were recorded after drug application in the two groups. VS was applied to left cervical vagus nerve when determine the parameters of ERP, WOV and AF inducibility in VS group.

#### Experiment 2

After drugs applied under the sub-epicardium at the PLA region, sixteen dogs were assigned into 2 groups: Group 1 (n = 8): atropine (1mM) application followed by RAP; Group 2 (n = 8): RAP followed by atropine (1mM) application. The ERP and WOV were determined from the electrodes of PV, PLA, LAR and LAA every hour during RAP in two groups.

### Statistical analysis

All data are reported as the mean ± SEM, one-way ANOVA was used for multiple comparisons of normally distributed data and Tukey's multiple comparison test was used for difference analysis between two groups (SPSS 16.0 software) in experiment 1. Coefficient of variation (COV) for each site was defined by ERP dispersion. In experiment 2, paired t-test was used for comparisons of ERP and WOV before and after drug application, ANOVA for repeated measurements was used for comparisons of ERP or WOV among different pacing hours and followed by post hoc testing for comparisons of the ERP and WOV at the end of each subsequent hour of pacing versus ERP and WOV in the baseline state. P <0.05 was considered significant.

## Results

The blood pressure was stable (118±26mmHg) during the whole period of experiments, and no evidence sign of heart failure was observed throughout the RAP period.

### Experiment 1

#### Atrial ERP and WOV

**Fig 2:**
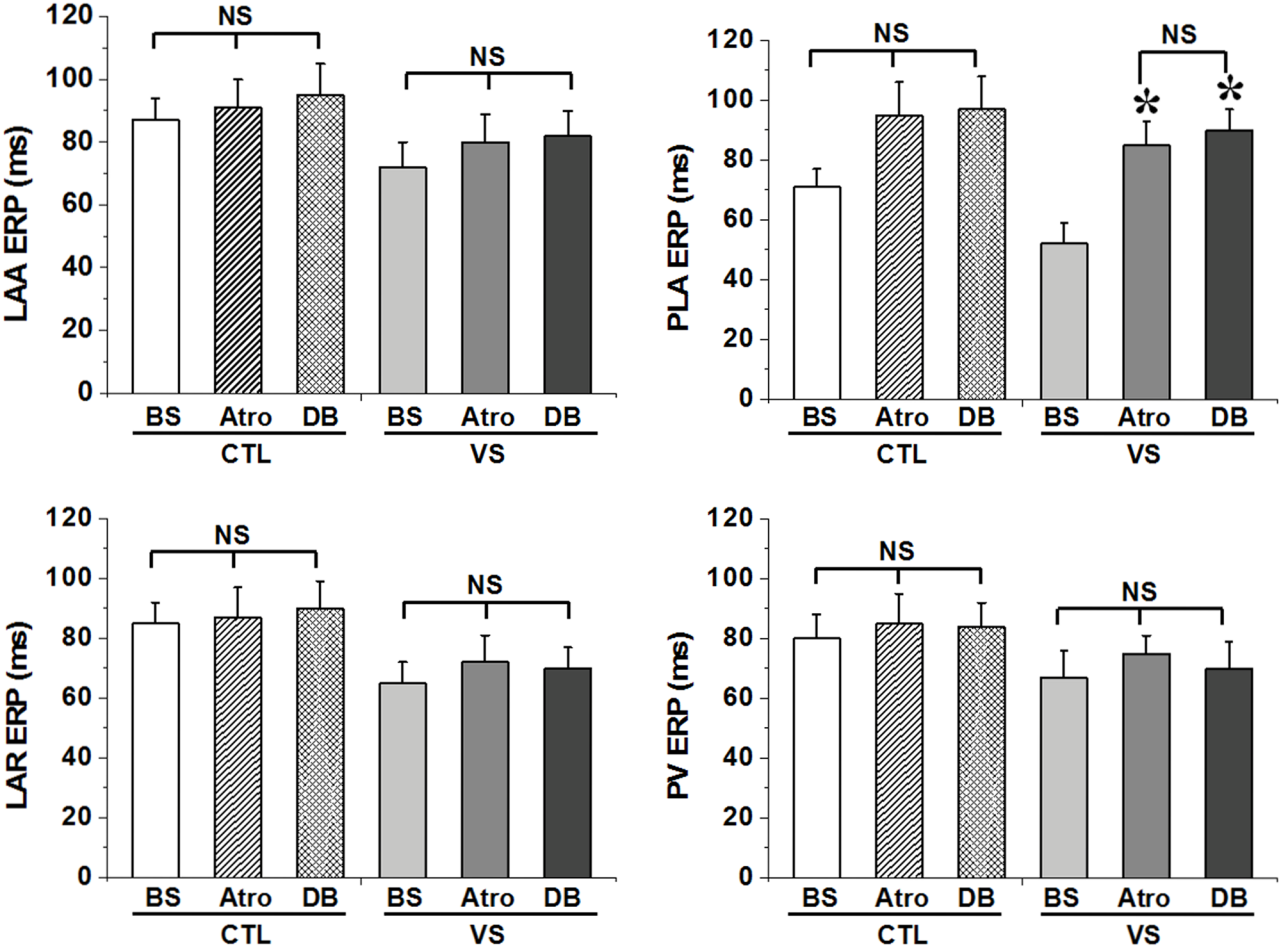
Effect of atropine alone and of double blockade of sympathetic and parasympathetic nerve (DB) with atropine and propranolol on effective refractory period (ERP) at each atrial and PV site, compared to baseline (BS) condition. There was a significant increase in ERP after drug application at PLA in vagus stimulation (VS) group. *P<0.05 for comparisons of the ERP after drugs application versus ERP in the BS in Tukey's multiple comparison test of ANOVA analysis; NS means no statistically significant by test of ANOVA analysis.

**Fig 3:**
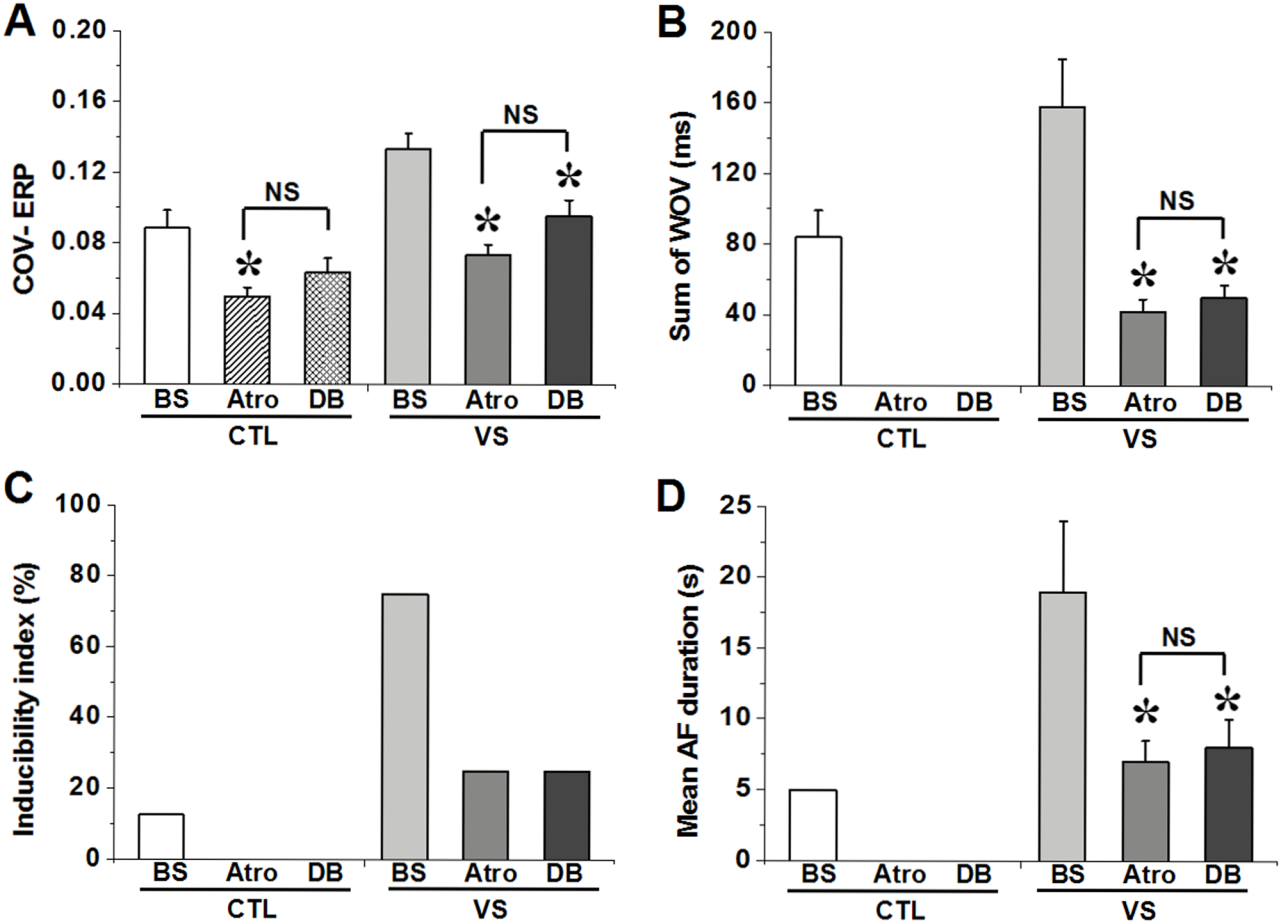
(A) Dispersion of ERP (COV-ERP) among atrial sites in control (CTL) and vagal stimulation (VS) groups. (B) Effect of drugs applied to PLA on sum of WOV. (C) inducibility index. (D) AF duration in control (CTL) and vagal stimulation (VS) groups. *P<0.05 for comparisons of the ERP after drugs application versus ERP in the BS by Tukey's multiple comparison test of ANOVA analysis; NS means no statistically significant by test of ANOVA analysis.

ERP was recorded at four sites in the left atrial chamber; stable ERP prolongation was observed at PLA after atropine administration. Fig 2 (Table A in S1 File) shows mean ERP at baseline and after vagal stimulation. Vagal stimulation led to more pronounced ERP shortening in PLA than in other sites (CTL:71±7 ms vs VS: 52±6 ms, P<0.05;); atropine alone and DB applied to PLA greatly inhibited this change (BS:52±6 ms vs Atro: 85±8 ms, P<0.05; BS:52±6 ms vs DB:90±9 ms, P<0.05). However, ERP did not differ significantly between atropine alone and DB conditions (P>0.05). The sinus rate was not significantly different before and 40min after administration of atropine (data not shown). ERP dispersion was significantly different between baseline and after vagal stimulation (P<0.01). However, VS induced ERP dispersion increase was markedly decreased by administration of atropine alone and DB (P<0.05) (Fig 3 and Table B in S1 File).

#### AF inducibility

AF inducibility measured by ƐWOV (sum of the WOV at each recording site), inducibility index and AF duration. At baseline, AF was rarely elicited using PES. However, AF inducibility was facilitated by vagal stimulation; over 60% of hearts (6/8) developed AF during PES. Moreover, AF duration and the sum of WOV from all atrial and PV sites were also significantly increased. After atropine application, AF inducibility was virtually inhibited, even in presence of VS. Only two episodes lasting >5 s could be elicited, and the ƐWOV markedly decreased to 42±7 ms after administration of atropine, compared with 158±27 ms in VS condition (P<0.05). However, double blockade had no additional effect on AF inducibility compared with atropine application alone (Fig 3 and Table B in S1 File).

#### AF characteristics

**Fig 4:**
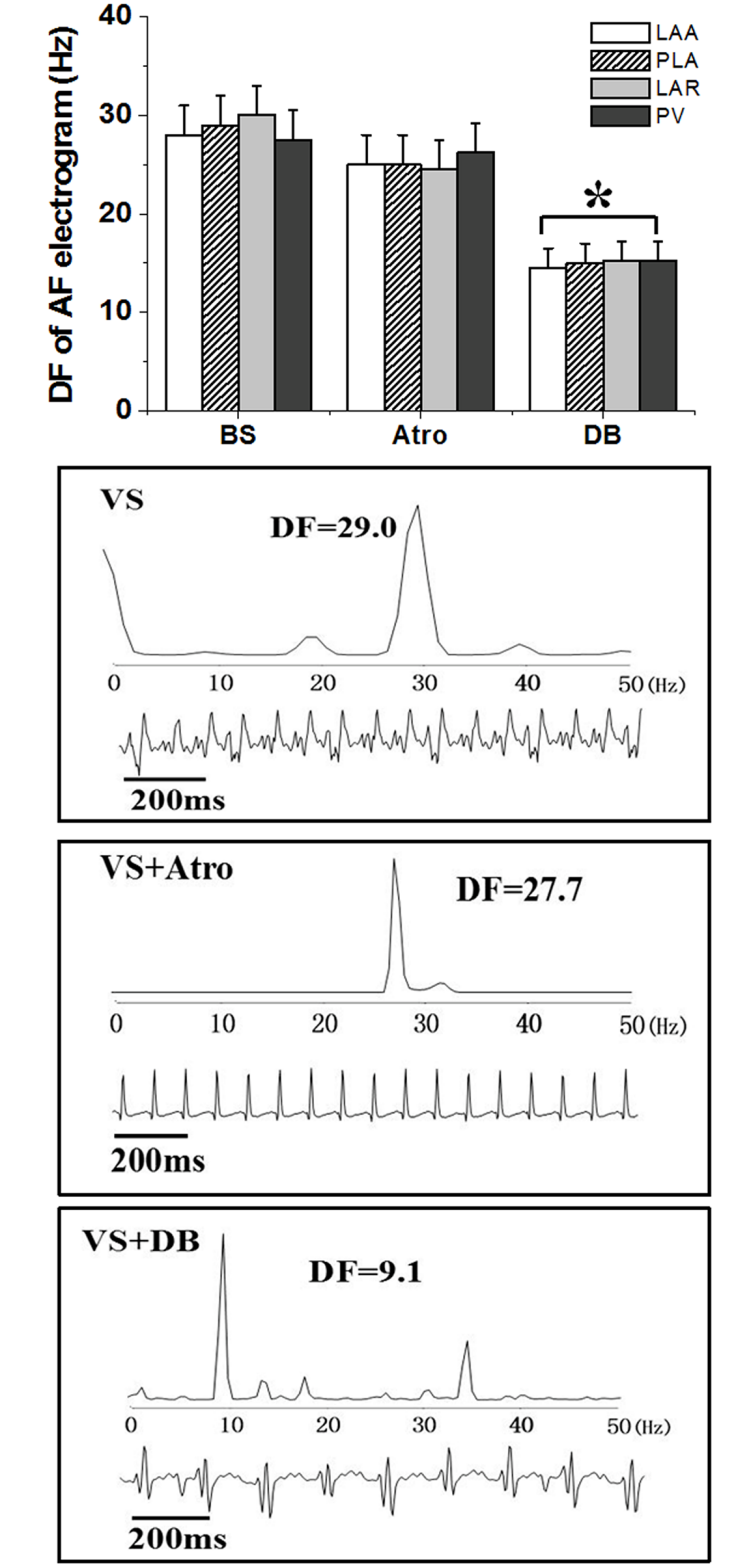
Frequency analysis of AF electrogram at each atrial site at baseline and after drug administration. Examples of PLA electrograms and corresponding power spectrum during baseline, atropine alone and DB conditions in VS group. *P<0.05 for comparisons of the ERP after drugs application versus ERP in the BS by test of ANOVA analysis.

Frequency analysis of AF electrograms showed that DF of VS-induced AF was not affected by atropine application. However, selective PLA double autonomic blockade with atropine and propranolol significantly decreased DF at all sites (P<0.05). Fig 4 (Table C in S1 File) shows DF for all sites before and after drug application.

### Experiment 2

#### ERP and ERP dispersion

**Fig 5:**
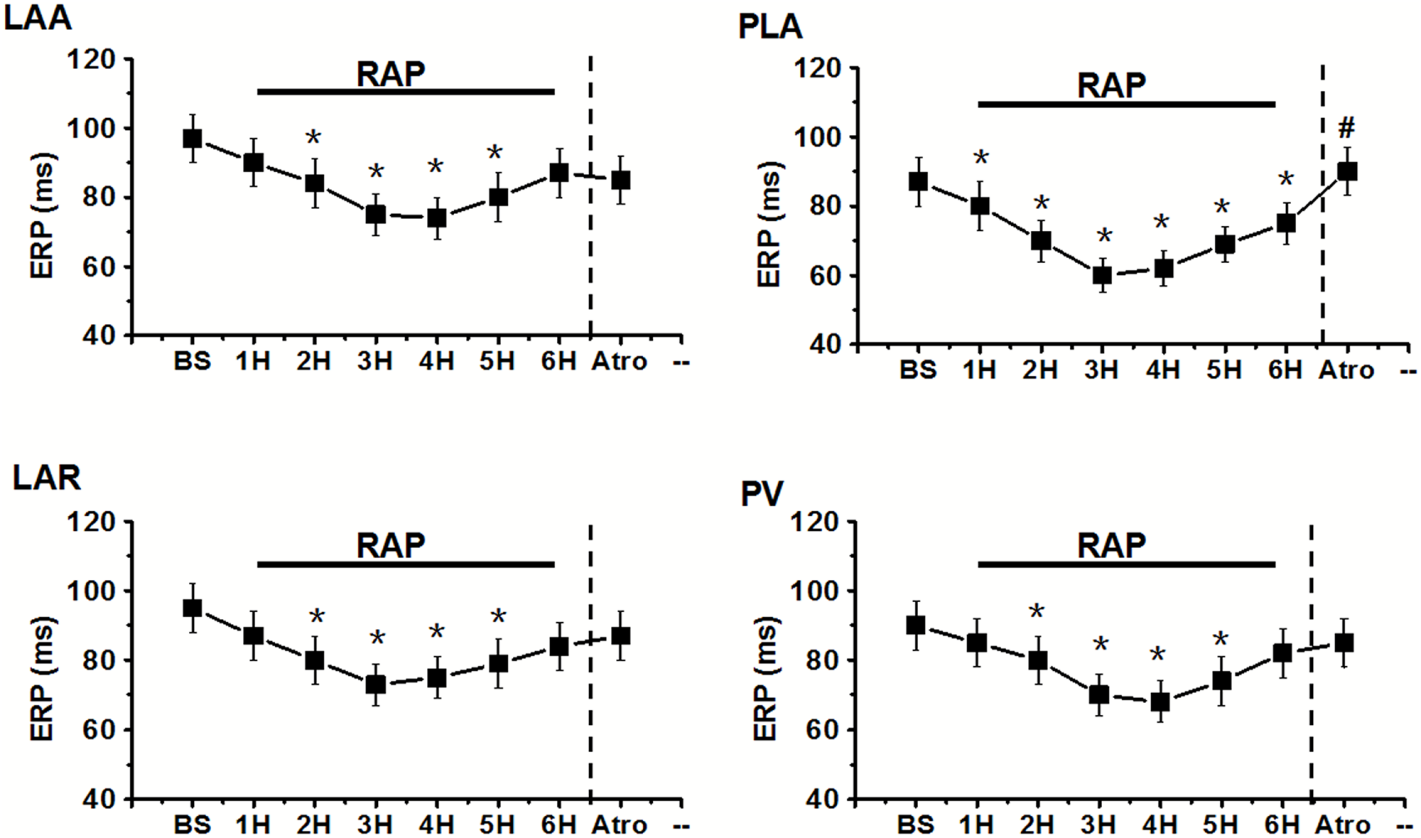
The changes in mean ERP at different recording sites during 6 hour RAP and after atropine application (Atro) in group 1. PV, pulmonary vein; PLA, posterior left atrium; LAR, left atrial roof; LAA, left atrial appendage. *P<0.05 for comparisons of the ERP at the end of each hour of pacing versus ERP in the baseline state (BS) by Tukey's multiple comparison test of ANOVA analysis; ^#^P<0.05 for comparisons of the ERP after drug application versus ERP immediately before drug application by Tukey's multiple comparison test of ANOVA analysis.

**Fig 6:**
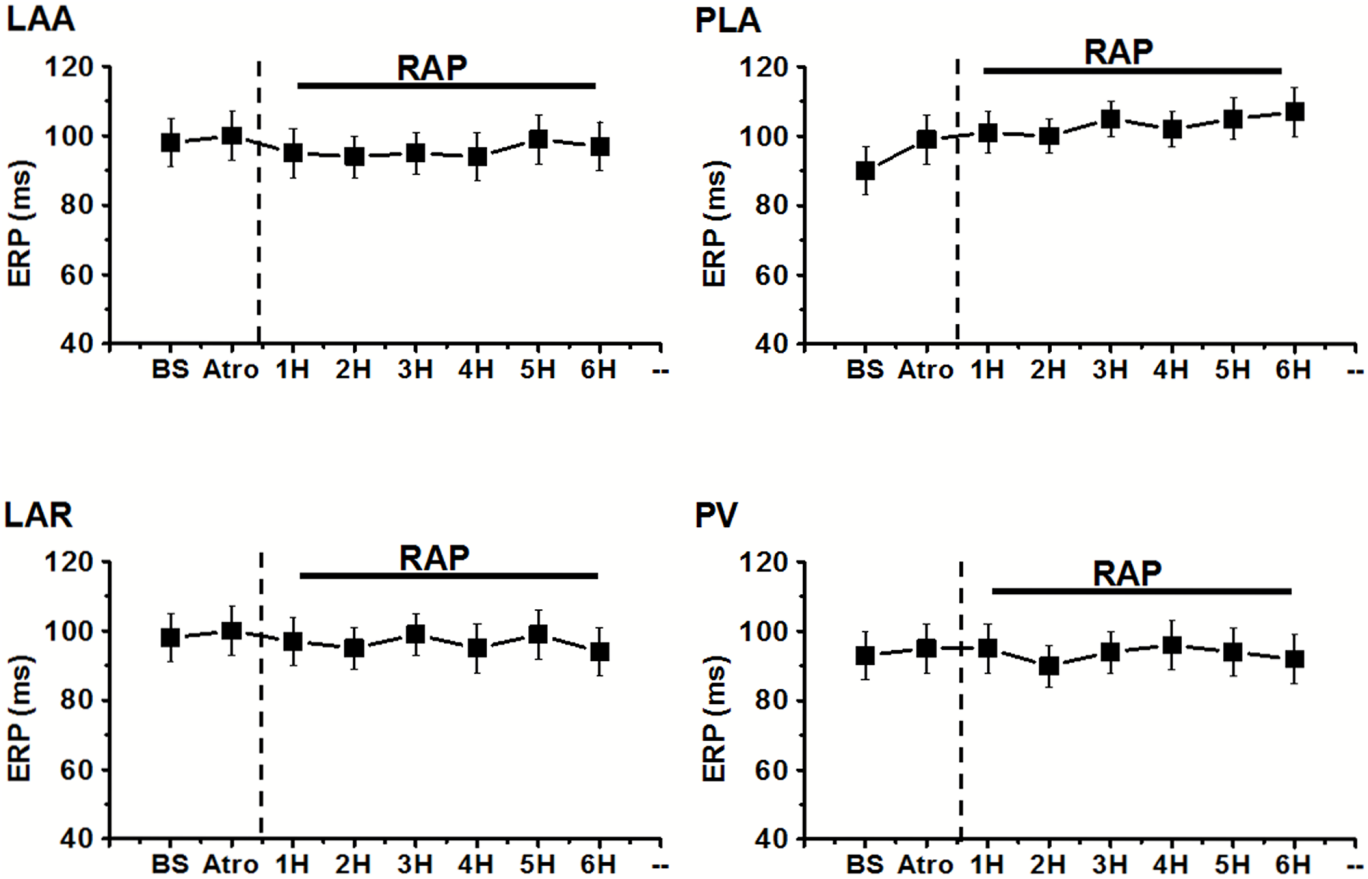
The changes in mean ERP at different recording sites after atropine application (Atro) followed by 6 hour RAP in group 2. *P<0.05 for comparisons of the ERP at the end of each hour of pacing versus ERP in the baseline state (BS) by Tukey's multiple comparison test of ANOVA analysis. PV, pulmonary vein; PLA, posterior left atrium; LAR, left atrial roof; LAA, left atrial appendage.

**Fig 7:**
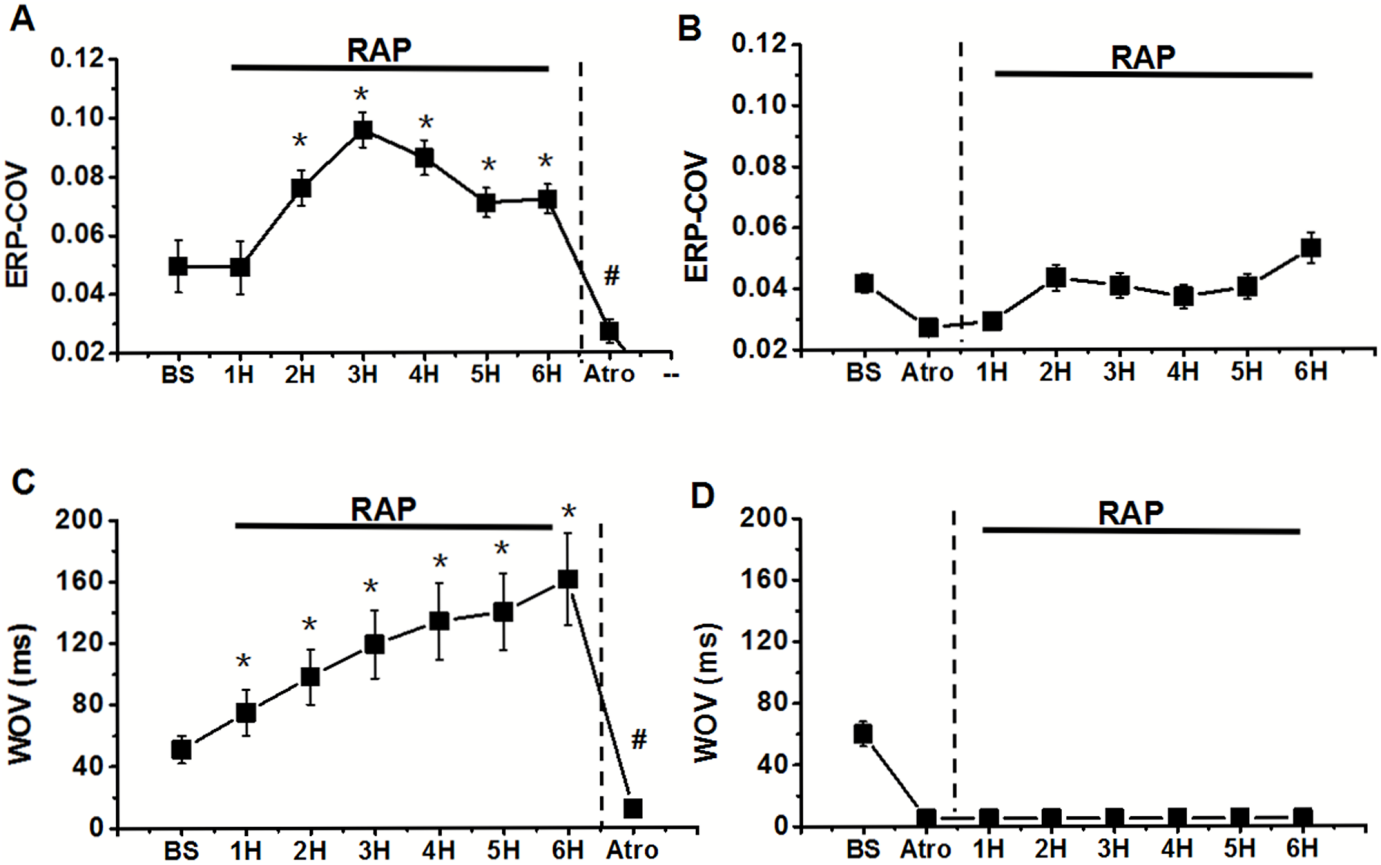
Changes of ERP-COV (A) and WOV (C) were calculated during RAP and after administration of atropine (Atro). And ERP-COV (B) and WOV (D) after autonomic blockers application followed by 6-hour atrial pacing were also examined. *P<0.05 for comparisons of the ERP at the end of each hour of pacing versus ERP in the baseline state (BS) by Tukey's multiple comparison test of ANOVA analysis; ^#^P<0.05 for comparisons of the ERP after drug application versus ERP immediately before drug application by Tukey's multiple comparison test of ANOVA analysis.

In group 1, the ERP of each site was significantly shortened after RAP from 2nd (11–19% decrease) to 6th hour (10–14% decrease) compared to baseline (BS) state, but it is failed to further decrease during periods of RAP from 4th to 6th hour (Fig 5 and Table D in S1 File). The ERP dispersion (ERP-COV) was also markedly increased at the second hour (2nd hour: 0.076±0.006 vs BS:0.050±0.09, P<0.05) and then stabilized from 2nd to 6th (6th hour: 0.072±0.04 vs BS: 0.050±0.09, P<0.05) hour after pacing. After administration of atropine in PLA, ERP was increased in all sites of left atria, but only the PLA showed the significant difference compared to the state before atropine application (90±8ms vs 76±6ms, P<0.05) (Fig 5 and Table D in S1 File). However, the increasing of ERP dispersion was greatly eliminated by using atropine (Atro: 0.027±0.002 vs 6th hour: 0.072±0.04, P<0.05). In group 2, after atropine application, RAP could not decrease the ERP at each site, and the ERP dispersion also failed to increase (Figs 6 and 7) at all (P>0.05).

#### WOV and AF inducibility

In group one, AF was elicited in all 8 dogs and the ƐWOV was progressively widened throughout the six hours pacing period (BS: 51±9ms vs 6th hour: 161±30ms, P<0.05). Especially, the WOV at the PLA markedly increased from 11ms in the BS state to 31ms at end of the RAP (data not shown). After administration of atropine, AF could be induced in only 1 of 8 dogs and the cumulative WOV was only 12ms (Fig 7C and Table F in S1 File). In group two, only 2 episodes of AF could be induced among all sites during RAP period (WOV = 0) (Fig 7D and Table G in S1 File).

## Discussion

In this study, we demonstrated that 1) autonomic response was more pronounced in the PLA than in other left atrial regions and PV; 2) PLA denervation greatly inhibited AF inducibility; 3) sympathetic and parasympathetic stimulation play different roles in AF genesis; 4) the RAP induced atrial electrical remodeling including shortening of ERP and increasing ERP dispersion could be prevented by PLA denervation; and 5) the inducibility of AF during RAP also be greatly inhibited by PLA denervation.

Vagal stimulation or administration of acetylcholine (Ach) have also been shown to decrease ERP but to increase ERP heterogeneity in the LA and enhance AF inducibility[1,2]. Moreover, evidence from clinical data suggested that vagal denervation enhanced benefit of circumferential pulmonary vein ablation in reducing AF recurrence [7]. However, the late breaking clinical trial (AFACT) [8] found that GP ablation during thoracoscopic surgery for advanced AF has no detectable effect on AF recurrence but causes more major adverse events. These contrary results may relate to the advanced nature of AF in patients of AFACT trial and autonomic remodeling (reinnervation and hyperactivity), occurring over the hierarchical gradient from the GP via the axonal field to the atrial neural network. Thus, ablation only at GP regions would be expected to be ineffective.

Previously, several studies demonstrated that both sympathetic and parasympathetic nerve densities decreased in the following order: PLA>PV>LAR>LAA[5,6,9]. Furthermore,VS caused greater decrease in ERP in PV and PLA, and the heterogeneity of vagal effects on refractoriness is more pronounced in the PLA as compared with other atrial sites[5]. The effects of VS on refractoriness are consistent with the relative anatomic profile patterns in these regions. As expected, Our approach using selective parasympathetic blockade in the PLA region led to more pronounced ERP increase and decreased ERP heterogeneity in the left atrium, and inhibited AF inducibility. Notably, the entire left atrial electrical remodeling could be prevented by PLA autonomic blockade alone, it suggested that the ERP shortening is due to the local release of autonomic neurotransmitters, especially acetylcholine, which enhanced to release in response to rapid atrial rate, confirming the presence of neural remodeling during RAP.

Neural remodeling is usually characterized by enhanced nerve activity and increased density of nerve fibers, M receptors and I_K,Ach_ channels[10–11]. It caused electrical remodeling may form a vicious cycle to initiating and sustaining the AF. Present observation suggested the effect of PLA denervation on neurotransmitters release in distal region of atria through the functional disruption of neural network in left atria during RAP, due to the presence of interconnections between the GP and nerve fibers in the atria[12–13]. Therefore, the remote effects of atropine on vagal responsiveness in the PV or other regions of left atria could be best explained by a positive feedback mechanism.

Sympathetic and parasympathetic stimulation have both been demonstrated to be proarrhythmic in the atrium. Vagal stimulation alone shortens atrial action potential duration and increases heterogeneity of atrial refractoriness. By contrast, sympathetic stimulation alone leads to a more homogeneous shortening of action potential duration[14–15]. Arora R et al demonstrated that parasympathetic nerve fibers are the predominant elements in PV, LAA, and especially in PLA[6]. Parasympathetic blockade significantly prolonged ERP and AF duration, and sympathetic blockade decreased conduction velocity. Thus double autonomic blockade had no additional effect on AF duration, instead it decreased dominant frequency of AF[16], supporting the notion that the parasympathetic system plays a dominant role in AF inducibility. Consistently, our results also showed that AF inducibility can be greatly inhibited by parasympathetic blockade in PLA, and double autonomic blockade only affected DF of AF episodes.

### Clinical implication

Previous studies in humans and animal models have suggested that PLA and PV are a dominant source of rapid activity for AF, secondary to particular autonomic nerve anatomic characteristics and myocardial structure[5,6,17]. However, cardiac denervation based on GP ablation showed lower success rate, likely because endocardial ablation strategies targeting the parasympathetic nerve are guided by nonspecific responses such as sinus bradycardia and AV block[3]. In addition, Tamborero et al reported that isolated PLA by adding linear lesions along the LA roof and connecting two inferior PVs did not improve the outcome of circumferential pulmonary vein ablation for AF[18]. Moreover, there is tissue damage and subsequent atrial tachycardia or complications after ablation. An ideal therapeutic approach would entail more precisely targeting the nerves involved in AF genesis. Our pharmacological approach targeting the innervation region appears to provide an effective way to terminate AF without any tissue damage, and it might become a novel strategy for AF prevention by using long-acting autonomic nerve system blocker.

### Study limitations

There are some limitations in the present study. Firstly, spectral analysis in animal and human AF demonstrated DF gradients toward the left and right atria, and significant increased DF in PLA compared to other region in persistent AF[19]. It is possible that the PLA contributed to the source of scroll wave for sustained AF. Thus, the drivers of arrhythmia need to be investigated by high density electrical mapping or high-resolution optical mapping to elucidate how the reentry mechanism is related to PLA denervation. Secondly, I_Na_ and I_Na-late_ current density in PLA myocytes has been reported to be significantly larger than that in LA anterior myocytes[20], which not only increases intracellular Na^+^ but also contributes to maintaining high intracellular Ca^2+^ in the PLA. These changes would lead to arrhythmogenesis by delayed after-depolarization in PLA. Therefore, beyond an autonomic mechanism, this underlying pro-arrhythmic effect might also contribute to AF inducibility. Thirdly, the burst pacing was not used in present study, although the burst pacing thought to be a non-physiological provocation, it will reliably induce AF via cardiac electrical instability[21]. Fourthly, The atrial remodeling in persistent AF was usually induced by long-term rapid pacing (4~6 weeks)[22]. These changes may lead to an “AF-begets-AF” vicious cycle and maintain the persistent AF. Therefore the effect of local denervation on persistent AF model and long-term atrial remodeling should be further examined.

## Conclusion

We conclude that blockade of the PLA could prevent genesis of AF and delayed the process of atrial remodeling induced by RAP. The autonomic profile of innervation region may be a crucial element in AF genesis and maintenance.

## Supporting information

S1 File Table A. Data of Fig 2. Table B. Data of Fig 3. Table C. Data of Fig 4. Table D. Data of Fig 5. Table E. Data of Fig 6. Table F. Data of Fig 7A and 7C. Table G. Data of Fig 7B and 7D.
(DOCX)
