# Supplementary material for: Neural substrate of posterior left atrium: A novel modulation for inducibility and remodeling of atrial fibrillation in canine
Source: PLoS One. 2017 May 5;12(5):e0176626. doi: 10.1371/journal.pone.0176626 (PMC5419517; doi:10.1371/journal.pone.0176626)
Supplement: S1 File — Table A. Data of Fig 2. Table B. Data of Fig 3. Table C. Data of Fig 4. Table D. Data of Fig 5. Table E. Data of Fig 6. Table F. Data of Fig 7A and 7C. Table G. Data of Fig 7B and 7D. (DOCX) [file pone.0176626.s001.docx]

|  | **CTL** | | | **VS** | | |
| --- | --- | --- | --- | --- | --- | --- |
|  | **BS** | **Atro** | **DB** | **BS** | **Atro** | **DB** |
| **LAA** | 87±7 | 91±9 | 95±10 | 72±8 | 80±9 | 82±8 |
| **PLA** | 71±7 | 95±11 | 97±11 | 52±6 | 85±8 | 90±9 |
| **LAR** | 85±7 | 87±10 | 90±9 | 65±7 | 72±9 | 70±7 |
| **PV** | 80±8 | 85±10 | 84±8 | 67±9 | 75±6 | 71±9 |

**S1 Table. Data of Figure2**

**S2 Table. Data of Figure3**

|  | **CTL** | | | **VS** | | |
| --- | --- | --- | --- | --- | --- | --- |
|  | **BS** | **Atro** | **DB** | **BS** | **Atro** | **DB** |
| **CoVERP** | 0.088±0.009 | 0.049±0.004 | 0.063±0.006 | 0.133±0.008 | 0.073±0.005 | 0.105±0.008 |
| **WOV(ms)** | 84±15 | 0 | 0 | 158±27 | 42±7 | 50±8 |
| **Index (%)** | 12.5 | 0 | 0 | 75 | 25 | 25 |
| **Duration(s)** | 5 | 0 | 0 | 19±5 | 7±2 | 8±2 |

**S3 Table. Data of Figure4**

|  | **LAA** | **PLA** | **LAR** | **PV** |
| --- | --- | --- | --- | --- |
| **BS** | 28±3 | 29±3 | 30±4 | 27.5±2.5 |
| **Atro** | 25±4 | 25.4±2.8 | 24.5±4 | 26.2±2.7 |
| **DB** | 14.5±2 | 15±2.1 | 15±1.8 | 15.2±2 |

**S4 Table. Data of Figure5**

|  | **BS** | **1H** | **2H** | **3H** | **4H** | **5H** | **6H** | **Atro** |
| --- | --- | --- | --- | --- | --- | --- | --- | --- |
| **LAA** | 97±7 | 90±7 | 84±8 | 75±6 | 74±7 | 80±8 | 87±8 | 85±7 |
| **PLA** | 87±7 | 80±8 | 70±6 | 60±5 | 62±5 | 69±6 | 76±6 | 90±8 |
| **LAR** | 95±8 | 87±8 | 80±8 | 73±6 | 75±7 | 79±9 | 84±8 | 87±8 |
| **PV** | 90±7 | 85±8 | 80±8 | 70±6 | 68±7 | 74±9 | 82±8 | 85±7 |

|  | **BS** | **Atro** | **1H** | **2H** | **3H** | **4H** | **5H** | **6H** |
| --- | --- | --- | --- | --- | --- | --- | --- | --- |
| **LAA** | 98±7 | 100±8 | 95±7 | 94±6 | 96±6 | 93±7 | 99±8 | 97±8 |
| **PLA** | 90±8 | 99±8 | 101±7 | 100±5 | 104±5 | 102±6 | 105±7 | 107±8 |
| **LAR** | 98±7 | 100±8 | 97±8 | 95±6 | 99±6 | 95±8 | 99±7 | 94±7 |
| **PV** | 93±7 | 95±8 | 95±7 | 90±6 | 94±6 | 96±7 | 94±7 | 92±8 |

**S5 Table. Data of Figure6**

**S6 Table. Data of Figure7 A and C**

|  | **BS** | **1H** | **2H** | **3H** | **4H** | **5H** | **6H** | **Atro** |
| --- | --- | --- | --- | --- | --- | --- | --- | --- |
| **COVERP** | 0.050±0.009 | 0.049±0.009 | 0.076±0.006 | 0.096±0.005 | 0.086±0.006 | 0.071±0.005 | 0.072±0.004 | 0.027±0.002 |
| **WOV** | 51±9 | 75±15 | 98±18 | 119±22 | 134±25 | 140±25 | 161±30 | 12 |

**S7 Table. Data of Figure7 B and D**

|  | **BS** | **Atro** | **1H** | **2H** | **3H** | **4H** | **5H** | **6H** |
| --- | --- | --- | --- | --- | --- | --- | --- | --- |
| **COVERP** | 0.042±0.003 | 0.024±0.002 | 0.029±0.002 | 0.043±0.003 | 0.041±0.003 | 0.037±0.002 | 0.040±0.004 | 0.053±0.002 |
| **WOV** | 60±8 | 0 | 0 | 0 | 0 | 0 | 0 | 0 |
